# Outcomes of surgically treated infective endocarditis in a Western Australian population

**DOI:** 10.1186/s13019-021-01727-0

**Published:** 2021-12-07

**Authors:** Aditya Eranki, Ashley R. Wilson-Smith, Umar Ali, Akshat Saxena, Eric Slimani

**Affiliations:** 1 Department of Cardiothoracic Surgery, John Hunter Hospital, Newcastle, Australia; 2 The Collaborative Research Group (CORE), Sydney, Australia; 3 Department of Cardiothoracic Surgery, Fiona Stanley Hospital, Perth, Australia

**Keywords:** Infective endocarditis, Valvular heart surgery, Cardiac Surgery, Mortality, Outcomes

## Abstract

**Background:**

Infective endocarditis is a disease that carries high morbidity and mortality. The primary endpoint of this study is to assess factors associated with in-hospital mortality in patients undergoing valvular surgery for infective endocarditis. The secondary endpoint of this study is to assess the incidence of post-operative stroke, renal failure, complete heart block and recurrence.

**Methods:**

Between the years of 2015 to 2019, a total of 89 patients underwent surgery for infective endocarditis at Fiona Stanley Hospital, Western Australia. Data was collected from the Australia and New Zealand Cardiac Surgery Database from 2015 to 2019 as well as patients electronic medical record. A number of preoperative and perioperative factors were assessed in relation to patient mortality and morbidity. Univariate and multivariate logistical regression analysis was done to assess for the association between factors and in-hospital morbidity and mortality.

**Results:**

A total of 89 patients underwent surgery for infective endocarditis from 2015 to 2019, affecting a total of 101 valves. The mean age of patients was 53.7 ± 16.5. A total of 79 patients had a positive blood culture pre-operatively, with *Staphylococcus Aureus* being the most frequently cultured organism (39%). Fourteen patients (16%) were deemed emergent and underwent surgery within 24 h of review. A total of five patients died within their hospital stay postoperatively. Variables significantly associated with mortality on univariate analysis were intravenous drug use, emergent surgery, perioperative dialysis, perioperative inotropes, cardiopulmonary bypass time and cross clamp time. Only CBP time was significantly associated with mortality on multivariate analysis. A total of 19 patients (21%) required hemodialysis after surgery, 10 patients sustained a postoperative stroke (11%), 11 patients developed a complete heart block post operatively (12%) and endocarditis recurred in 10 patients (11%).

**Conclusion:**

Prolonged cardiopulmonary bypass times were significantly associated with mortality. This study is novel to report a lower mortality rate than previously quoted in the literature. We also report our findings of organisms, preoperative embolic phenomena and surgery in a Western Australian population. We recommend that all patients with endocarditis are discussed in multidisciplinary forum.

## Background

Infective endocarditis (IE) is associated with a high morbidity and mortality, with the mortality rate in studies ranging between 6 and 25% [1–5]. Patients can suffer from complications such as embolization, stroke, heart failure and cardiogenic shock, disseminated infection, abscess formation and arrythmias, including complete heart block. Factors associated with these complications have also been assessed in literature, although to a lesser extent [2–14]. Emergent surgery is often required, ranging between 25 and 50% of these cases [4, 7]. The indications for early or emergent surgery in IE has been outlined recently by the European Society of Cardiology, with cardiogenic shock, high-risk vegetations and locally uncontrolled infection being the primary factors considered [2]. Grey areas exist with regards to timing of surgery in the setting of a pre-operative embolic stroke [2]. The concept of a ‘Heart Team’ comprising of various specialties to create management plans for individual cases has been shown to decrease mortality in these cases [2]. In this report, we describe our experience of surgically treated endocarditis in a single Australian institution with a comprehensive overview of organisms, sites of embolization, operation performed and surgical outcomes. The primary outcome of this study is to assess factors associated with in-hospital mortality in patients with IE undergoing surgery. The secondary outcome of this study is to assess the factors associated with morbidity; namely post-operative stroke, renal failure and dialysis, complete heart block and recurrence.

## Methods

Data was retrospectively collected from the Australian and New Zealand Cardiac Surgery database (ANZSCTS). Between the years of 2015 to 2019, a total of 89 patients underwent surgery for IE at Fiona Stanley Hospital. Cases of IE related to cardiovascular devices were excluded. Furthermore, cases of infective endocarditis were retrospectively confirmed using the modified Dukes Criteria and patients that did not meet these criteria were excluded [15]. A total of 9 patients were excluded. Preoperative and operative factors were identified and recorded through a combination of the ANZSCTS database and patient electronic medical record (EMR). These factors were selected as they are assumed to be associated with an increased rate of morbidity and mortality post-surgery. The definitions of these factors are in accordance with those set by the ANZSCTS database. The valvular procedure was documented from a combination of the ANZSCTS database and the patient electronic medical record. This includes whether the patient had an aortic procedure, aortic valve, mitral valve, tricuspid valve or pulmonary valve procedure. The number of valves affected was documented, as well as whether the index operation was a valvular replacement or repair. In valve replacements, the prosthesis type was recorded (mechanical or bioprosthetic). In patients who underwent valvular repair, details of the repair were recorded.

### Outcomes

The primary endpoint was defined as 30-day mortality. The second endpoint was the incidence of, and factors associated with post-operative sequelae: cerebrovascular accident (CVA), post-operative hemodialysis, new complete heart block (CHB) and recurrence. CVA post-operatively was defined as the occurrence of a stroke or new central neurologic deficit (persisting > 72 h) post-operatively. Recurrence was defined as a new diagnosis of endocarditis after the patient’s index operation for endocarditis.

### Statistical analysis

Descriptive statistics, including number of events, mean and standard deviation, were calculated for each preoperative and operative variable. Univariate analysis was conducted to identify variables significantly associated with either in-hospital mortality or morbidity. Categorical variables were assessed using the chi-squared (x^2^) test to ascertain odds ratios. Fishers exact test was conducted when more than 20% of cells on the contingency table had an expected frequency less than five. Continuous variables were first assessed for normality and then with the independent *T* test to assess for equality of means. Variables that were not normally distributed were assessed with the Mann–Whitney U test. *P* values less than 0.05 were deemed as significant. Preoperative and operative variables that reached significance were then further assessed with multivariate logistic regression analysis using a binary logistic regression model. Statistical analysis was done on IBMM ® SPSS statistics version 25.

## Results

### Demographic data

A total of 89 patients underwent surgery for IE, affecting 101 valves. The mean age of patients was 53.7, with a minimum age of 16 and a maximum age of 83. Most of the patients were male (n = 67). A large portion of patients (n = 28) had a history of IVDU. Seventeen patients had a history of Diabetes. Fifteen patients identified as ATSI.

### Preoperative patient factors

A total of 15 patients (17%) were in cardiogenic shock perioperatively. Eleven patients (12%) required perioperative hemodialysis, 20 patients (22%) were in perioperative respiratory failure and 17 patients (19%) had perioperative inotrope requirements*.* Twenty-seven patients had vegetations greater than 20 mm on echocardiography, 32 patients had vegetations between 10 and 20 mm and 30 were less than 10 mm. Most patients who underwent surgery had left-sided disease (82 patients) with 7 patients undergoing surgery for right-sided disease (see Table 1). A total of 79 patients had a positive blood culture pre-operatively, with *Staphylococcus Aureus* being the most cultured organism (39%). Ten patients (11%) had culture negative endocarditis. These results are summarized in Table 2. Embolic phenomena were present in 39 patients (44%). The most common site of embolization noted was the brain, noted in 23 patients (26%). Other common sites were skin (6 patients), lungs (5 patients) and spine (4 patients). Ten patients had multiple sites of embolization. These results are summarized in Table 3.

**Table 1:** Preoperative and operative variables

Preoperative variable	Preoperative variables	Operative variables
*Age*	*Preoperative cardiogenic shock*	*Cardiopulmonary bypass time*
53.7 years	Yes 15	137 min
	No 74	
*Gender*	*Preoperative dialysis*	*Cross clamp time*
Male 67	Yes 11	102 min
Female 22	No 78	
*IVDU history*	*Preoperative respiratory failure*	*Aortic procedure*
Yes 28	Yes 20	Yes 13
No 61	No 69	No 76
*Location*	*Preoperative inotrope requirement*	*Double valve replacement*
Rural 33	Yes 17	Yes 8
Metropolitan 56	No 72	No: 81
*ATSI*	*Urgency*	*Mitral valve repair*
Yes 15	Elective 13	Yes 15
No 74	Urgent 62	No 28 (replacement)
	Emergent 14	
*Diabetes*		
Yes 17		
No 72		
*BMI*		
27.8		
*NYHA status*		
NYHA 1 52		
NYHA 2 11		
NYHA 3 13		
NYHA 4 13		
*Positive blood culture*		
Yes 79		
No 10		
*Organism*		
Staph A: 35		
Other: 54		
*Vegetation size (mm)*		
0–10: 30		
10–20: 32		
> 20: 27		
*Native or prosthetic valve infection*		
Native valve 68		
Prosthetic valve 21		
*Left sided disease*		
Yes 82		
No 7		
*Preoperative embolic event*		
Yes 39		
No 50		
*Cerebral Emboli*		
Yes 23		
No 66		
*Preoperative creatnine*		
134 micromol/L

**Table 2:** Organisms isolated

Organism (N = 79)	Number (%)
*Staphylococcus Aureus*	35 (29%)
*Enterococcus Faecalis*	18 (20%)
*Streptococcus Mitis*	8 (9%)
*Streptococcus Mutans*	3 (3.3%)
*Streptococcus Anginosus*	2 (2.2%)
*Streptococcus gordonii*	2 (2.2%)
*Escherichia Coli*	2 (2.2%)
*Streptococcus Agalactiae*	2 (2.2%)
*Streptococcus Parasanguinis*	1 (1.1%)
*Streptococcus Sanguinis*	1 (1.1%)
*Staphylococcus Hominins*	1 (1.1%)
*Capnocytophaga Canimorsus*	1 (1.1%)
*Streptococcus Pneumonia*	1 (1.1%)
*Staphylococcus Epidermitis*	1 (1.1%)
*Streptococcus Oralis*	1 (1.1%)
*Haemophilus Influenzae*	1 (1.1%)
*Neisseria Gonorrhoeae*	1 (1.1%)
*Aerococcus Urinae*	1 (1.1%)
Culture negative	10 (11%)

**Table 3:** Sites of embolic phenomenon

Embolic phenomenon (N = 39^a^)	Number
Lungs	5
Spleen	2
Spine	4
Joints (septic arthritis)	2
Brain	23
Skin	6
Vascular	3
Kidney	1
Other	4

^a^10 patients had multiple sites of embolization

### Operative data

Fourteen patients (16%) were deemed emergent and underwent surgery within 24 h of diagnosis, whereas the majority of patients (62) underwent urgent surgery (within 72 h). The mean cardiopulmonary bypass time was 137 min, and the mean cross clamp time was 102 min. The aortic valve was most affected, with 23 patients undergoing a mechanical aortic valve replacement (AVR) and 29 undergoing a tissue AVR. The mitral valve was also frequently involved, with 18 patients receiving a mechanical mitral valve replacement (MVR) and 10 receiving a tissue MVR. We opted to repair the mitral valve in 15 patients (35%). One patient underwent a mechanical tricuspid valve replacement (TVR) and one patient underwent a bioprosthetic pulmonic valve replacement. Not uncommonly, infection involved the aortic root, with 11 patients undergoing a Bentalls procedure (12%). Eight underwent double valve replacement, seven of which received an AVR and MVR and one patient receiving an AVR and pulmonic valve replacement (see Table 4).

**Table 4:** Valve and procedure

Procedure/prosthesis	Aortic valve (N = 52)	Mitral valve (N = 43)	Tricuspid valve (N = 9)	Pulmonary valve (N = 1)
Mechanical	23	18	1	
Bioprosthetic	29	10		1
Bentalls	11			
Hemiarch	1			
Annuloplasty		12	5	
Suture repair		5	3	
Neochords		8		
Commisuroplasty		1		
Other	2^a^			

^a^One patient: aortic root suture repair, one patient: resection subaortic membrane

### Primary endpoint: factors affecting in-hospital mortality

Five patients died within 30 days of their index operation (6%). Variables associated with mortality on univariate analysis were IVDU (OR 10, *P* = 0.032), emergent surgery (OR 9.95, *P* = 0.026), preoperative dialysis (OR 44, *P* = 0.001), preoperative inotropes (OR 21.8, *P* = 0.004), CPBT with 250.8 min for non-survivors compared to 130.5 min for survivors (*P* < 0.001) and cross-clamp time (CCT) with 175 min for non-survivors compared to 97.8 min for survivors (*P* = 0.006). Multivariate analysis revealed that only CBP time was a significant predictor of operative mortality, with an odds ratio of 1.05 per minute of additional bypass time (95% CI 1.001–1.101, *P* = 0.046). These results are outlined in Table 5*.* A further subgroup analysis was performed on factors associated with prolonged CPBT, demonstrating that prosthetic valve involvement, *Staphylococcus Aureus* infection and aortic surgery were significantly associated with prolonged CPBT. Aortic surgery was significant on multivariate analysis (OR 27.8, 95% CI 3.94–200). This is outlined in Table 6.

**Table 5:** Outcome 1—univariate analysis of in hospital mortality

Variable assessed	Death (N = 5)	Survivors (N = 84)	Odds ratio	*P* value	Adjusted OR
Age	52.4	53.8		*P* = 0.852	
Gender (male)	4	63		*P* > 0.900	
IVDU	4	24	10.0 (1.0–94)	*P* = 0.032	
Rural patient	3	30		*P* = 0.355	
ATSI	2	13		*P* = 0.196	
Hx of diabetes	1	16		*P* > 0.900	
BMI	28.0	27.7		*P* > 0.900	
NYHA 4	2	11		*P* = 0.153	
Positive blood culture	4	75		*P* = 0.457	
Staph A	3	32		*P* = 0.378	
Vegetation > 20 mm	3	24		*P* = 0.161	
Prosthetic valve involved	3	18		*P* = 0.083	
Left sided endocarditis	5	77		*P* > 0.900	
Embolic phenomenon	2	37		*P* > 0.900	
Cerebral emboli	1	22		*P* > 0.900	
Preoperative creatinine	118	135		*P* = 0.624	
Emergent surgery	3	11	9.95 (1.49–66.4)	*P* = 0.026	
Preoperative cardiogenic shock	2	13		*P* = 0.196	
Preoperative respiratory failure	3	17		*P* = 0.073	
Preoperative dialysis	4	7	44.0 (4.31–449)	*P* = 0.001	
Preoperative inotropes	4	13	21.8 (2.26–211)	*P* = 0.004	
Aortic procedure	2	11		*P* = 0.153	
Double valve replacement	1	10		*P* = 0.602	
Mitral valve repair	0	14		*P* = 0.320	
CPB time	250.8	130.5	1.02 (1.01–1.03)	*P* < 0.001	OR 1.05 (1–1.1)
Cross clamp time	175	97.8	1.26 (1.01–1.04)	*P* = 0.006

**Table 6:** Prolonged CBPT

Variable	Odds ratio	*P* value	Adjusted OR
Prosthetic valve endocarditis	N = 8 OR 3.19 (CI 1.07–9.50)	*P* = 0.032	
Staph A	N = 12 3.50 (1.22–10.1)	*P* = 0.016	
Aortic procedure	N = 9 OR 14.9 (3.84–57.4)	*P* < 0.001	27.8 (3.94–200)

### Secondary endpoint: factors affecting postoperative morbidity

Nineteen patients (21%) required hemodialysis after surgery. Perioperative cardiogenic shock and perioperative dialysis were significantly associated with post-operative dialysis, with an odds ratio of 9.35 (95% CI 1.47–58.8, *P* = 0.018) and 20 (95% CI 2.24–167, *P* = 0.007) respectively. A total of 10 patients sustained a postoperative CVA (11%). A number of factors were significantly associated with postoperative stroke, however, none of these reached significance on multivariate analysis. Eleven patients developed a CHB post-operatively. All these patients required a pacemaker insertion. Factors associated with the development of CHB was cross-clamp time (130 min vs 98 min *P* = 0.036) and whether the patient had an aortic procedure (OR 4.38, *P* = 0.05). Infective endocarditis recurred in a total of 10 patients. Early recurrence (within a year of the index operation) occurred in four patients. None of these factors were significantly associated with recurrence. These results are outlined in Table 7.

**Table 7:** Outcome 2—univariate analysis of morbidity

Variable	Postoperative stroke (N = 10)	Postoperative dialysis (N = 19)	Recurrence (N = 10)	Complete heart block (N = 11)
NYHA 4		N = 7 OR 6.22 (1.8–22) *P* = 0.005		
Preoperative emboli	N = 8 OR 6.19 (1.23–31) *P* = 0.019			
Cerebral emboli	N = 6 OR 5.47 (1.38–22) *P* = 0.017			
Preoperative creatinine	207 versus 125 *P* 0.036			
Preoperative cardiogenic shock	N = 5 OR 6.90 (1.7–28) *P* = 0.011	N = 10 OR 14.4 (4.0–52) *P* < 0.001		
Preoperative respiratory failure	N = 5 OR 4.26 (1.1–16) *P* = 0.042	N = 10 OR 6.67 (2.2–20) *P* = 0.001		
Preoperative ionotropes	N = 5 OR 5.58 (1.4–22) *P* = 0.020	N = 11 OR 14.7 (4.3–51) *P* < 0.001		
Preoperative dialysis		N = 9 OR 30.6 (5.8–162) *P* < 0.001		
Emergent procedure	N = 4 OR 4.60 (CI 1.1–19) *P* = 0.047	N = 8 OR 7.76 (2.3–27) *P* = 0.001		
Cross clamp time (min)				130 versus 98 *P* = 0.036
RBC transfusion		N = 17 OR 7.16 (1.5–33) *P* = 0.007		
Aortic procedure				N = 4 OR 4.38 (1.1–18) *P* = 0.05

## Discussion

Surgical treatment for IE is associated with a high mortality rate, quoted between 6 and 25% [3, 4, 7–12]. Risk factors associated with mortality include older age, emergent surgery, septic shock, congestive heart failure, cardiogenic shock, high risk organisms, prosthetic valve infection and stroke [1, 3–12, 16]. The European Society of Cardiology (ESC) provide guidelines for the management of IE [2]. The guidelines advocate for early surgery in patients with heart failure, uncontrolled infection and high-risk lesions to prevent embolization [2]. Of all factors, congestive cardiac failure is the most consistent predictor of mortality [17, 18]. These studies advocate for early surgery in patients presenting in heart failure [17–19]. Early surgery for high-risk lesions is also supported by literature [20–22]. Of these, a randomized control trial by Kang et al. [21] demonstrated that early surgery in patients with large left-sided lesions (> 10 mm) significantly reduced morbidity and embolic events. The ESC guidelines provide a class 1 indication for early surgery in vegetations greater than 10 mm with ongoing embolic phenomena. Uncontrolled infection is a further indication for early surgery. This is supported by several retrospective cohort studies, demonstrating that locally aggressive infection is associated with a higher mortality rate [10, 23].

Of these, a retrospective study by Revilla et al. [10] demonstrated that persistent infection is an independent predictor of mortality, where patients who undergo urgent surgery with persistent infection are four-fold as likely to die as patients without persistent infection. At Fiona Stanley hospital, we adopted these guidelines to help with decision making regarding operative timing. In the current study, the in-hospital mortality rate was 5.6% or 5 out of 89 patients. This finding is novel as it is at the lower end of the spectrum of mortality figures quoted by other studies [3, 4, 6, 9, 10]. *Rivas de Oliveira* assessed 88 surgical patients between 2005 and 2015 and reported an in-hospital mortality rate of 17% [3]. Dunne et al. [11] in a similar Western Australian population with IE reported a mortality rate then of 13%. One major change reported amongst hospitals during the last decade is the establishment of a dedicated “heart team”. This team comprises of Cardiac Surgeons, Cardiologists and Infectious Diseases physicians. A dedicated “heart team” was established at Fiona Stanley Hospital since its initiation in 2015. Studies have reported a decline in mortality as a result of a multidisciplinary team (MDT) approach to endocarditis [24, 25]. A retrospective study by Chirillo et al. [24] demonstrated that after the implementation of an MDT, in-hospital mortality reduced from 28 to 13%, as well as surgical mortality from 47 to 13%. Similarly, a retrospective study conducted by Botelho-Nevers et al. [25] identified that MDT approach to endocarditis yielded a significant decrease in 1-year mortality, from 18.5 to 8.2%. There was also a statistically significant increase in compliance to antimicrobial therapy. The 2015 ESC guidelines (class 2 evidence) recommend the timing of surgical intervention via the consensus of an MDT team [2]. Our practice at Fiona Stanley Hospital is to conduct weekly MDT meetings to discuss cases of endocarditis which has potentially contributed to the low mortality rate.

Our study identified that IVDU, emergent surgery, perioperative dialysis, perioperative inotropes, prolonged cardiopulmonary bypass (CPB) time and prolonged CCT were significantly associated with in-hospital mortality on univariate analysis. This finding is consistent with previous studies [1, 3, 6, 11, 26]. CPB time was the only factor to be significantly associated with death on multivariate analysis, with a mean CBP time of 250.8 vs 130.5 min for non-survivors and survivors respectively. A further analysis demonstrated that prosthetic valve involvement, *Staphylococcus Aureus* infection and aortic surgery was significantly associated with prolonged CPBT with aortic surgery reaching significance on multivariate analysis. Prolonged CPB time is a reflection of operative complexity, predisposes patients to end organ dysfunction, coagulation disorders and is therefore understandably associated with mortality.

Embolic phenomena occurred 39 patients (43.8%). The most common site of emboli was the brain (22 patients) followed by skin and lungs. Other studies have also quoted equally high rates of embolic events [10, 27]. Likewise, in these studies, the brain was the most common site of embolism [10, 27]. Pre-operative stroke is a highly relevant complication of IE due to the risk of hemorrhagic transformation and postoperative neurological deterioration. Guidelines provide class 2A evidence to delay surgery by a month in the presence of intracranial haemorrhage [2]. As a result, we adopted a low threshold to conduct a CT brain, explaining the higher rate of cerebral emboli compared to other sites in this study. Embolic phenomena and cerebral emboli were linked to the incidence of preoperative stroke on univariate analysis, however, was not associated with in-hospital mortality.

In terms of organisms, *Staphylococcus Aureus* was most cultured and present in 39% of patients. This was followed by *Enterococcus Faecalis* and *Streptococcus Mitis* in 20% and 9% of patients respectively. Eleven percent of patients had culture negative IE. The prevalence of *Staphylococcus Aureus* is a feature in other studies also [6, 10]. There has been a reported shift in the epidemiology of IE away from *Streptococcus* and HACEK (Haemophilus species, Aggregatibacter species, Cardiobacterium hominis, Eikenella corrodens and Kingella) organisms towards *Staphylococcus Aureus *[28, 29]*.* This was also evident in our study, with only 15 patients culturing Viridians Streptococci. There was one case of HACEK endocarditis. *Staphylococcus Aureus* has been linked to a higher mortality rate in surgically treated endocarditis [30, 31]. It is also linked to locally aggressive infection, higher rates of embolization and septic shock [30, 31]. Our study did not demonstrate a relationship between *Staphylococcus Aureus* and in-hospital mortality or post-operative complications, however, we did demonstrate that may be linked with prolonged CPBT and operative complexity. At our institution, we favour early surgery for patients with *Staphylococcus Aureus* endocarditis.

The majority of our patients received a valve replacement. This was especially the case with aortic valve endocarditis, where all patients received a valve replacement. We opted to repair the mitral valve in 14 cases (33%). The rate of repair is consistent with that reported in literature [32]. Mitral valve repair is associated with lower in hospital mortality and morbidity in literature, however, this was not reported in our study [32]. Twelve patients had endocarditis of the aortic root with periannular abscess formation. In cases such as this, we opted to perform radical debridement of the annulus followed by replacement of the aortic valve and root. In our centre, we opted to use a valved graft conduit in a Bentalls procedure, however, some studies advocate for allografts as they demonstrate a lower rate of postoperative graft infection [33]. Aortic surgery in endocarditis is associated with a high morbidity and mortality [34]. In our study, it was not significantly associated with mortality, though it was associated with longer CPBT and postoperative CHB. Surgery for right-side endocarditis was uncommon and was performed in 10 patients (11%). Only four patients had isolated tricuspid valve replacements. All other cases of right-side disease were performed in conjunction with left-side valve surgery. One patient underwent a pulmonic valve replacement. Surgery for pulmonic valve endocarditis is rare and is most commonly performed on prosthetic infections of pulmonic valve allografts (Ross procedure) or in conjunction with other valves [35]. It is unusual to be performed in isolation [35]. Studies report excellent short- and long-term outcomes despite being an uncommon pathology [35]. In our case, it was performed with concurrent AVR.

Complications after surgery for IE were not uncommon. Ten patients (11%) had a postoperative stroke. Identifiable risk factors were cerebral emboli, pre-operative creatinine, perioperative cardiogenic shock, perioperative respiratory failure, perioperative ionotropic requirement and emergent procedure. Other studies have demonstrated a similar incidence of post-operative stroke [10, 11, 27]. Only one other study investigated risk factors associated with post-operative stroke [11]. Post-operative stroke is a debilitating issue, and some centers advocate for delaying surgery to minimize the risk of hemorrhagic transformation [22, 31]. Others demonstrate that the overall mortality benefit from early surgery outweighs this risk [36]. The practice at Fiona Stanley Hospital was to delay surgery by a month if feasible if there is a significant risk of hemorrhagic transformation. A total of 19 patients (21%) required dialysis postoperatively. On multivariate analysis, cardiogenic shock and pre-operative dialysis were independently associated with the incidence of post-operative dialysis. Post-operative renal failure is linked to a critical perioperative state and is associated with an increased risk of mortality [6, 10, 37, 38]. Conduction abnormalities are an early indication of an infectious process expanding to involve the membranous interventricular septum, often in cases with aortic valve endocarditis. A total of 11 patients (12%) had complete heart block, all of whom received a pacemaker. The incidence of which is comparable to that published in other studies [6, 39].

This is a retrospective observational study with inherent biases in data collection. A larger prospective study may enable us to explore more factors associated with morbidity and mortality. Our small patient numbers and the small number of in-hospital deaths have limited the use of multivariate analysis to evaluate risk factors for in-hospital mortality. Fiona Stanley Hospital is a new institution, and data is available over a period of 4 years. As a result, long term morbidity and survival data was not explored by this study and therefore Kaplan–Meier survival analysis was not conducted. Long term follow-up of our patients would be beneficial to assess whether the low in-hospital mortality rate is also translates into long term survival.

## Conclusion

This study reports the morbidity and in-hospital mortality of 89 patients undergoing valvular surgery for IE at a single institution. Prolonged CPBT is significantly associated with mortality. Our study is novel in its reporting of a low 30-day mortality rate and exemplifies the need for a multidisciplinary approach to the management of endocarditis.

## Data Availability

The datasets used and/or analysed during the current study are available from the corresponding author on reasonable request.

## Abbreviations

IE: Infective endocarditis
EMR: Electronic medical record
CCT: Cross clamp time
CPBT: Cardiopulmonary bypass time
ANZSCTS: Australia and New Zealand Cardiac Surgery
IVDU: Intravenous drug use
ATSI: Aboriginal and Torres Strait Islander
BMI: Body mass index
NYHA: New York Heart Association
RBC: Red blood cell
NRBC: Non red blood cell
CVA: Cerebrovascular accident
AVR: Aortic valve replacement
MVR: Mitral valve replacement
TVR: Tricuspid valve replacement
ESC: European Society of Cardiology
MDT: Multidisciplinary team
